# 4-(2-Bromo­phen­yl)-2-phenyl­pyrano[3,2-*c*]chromen-5(4*H*)-one

**DOI:** 10.1107/S1600536812044510

**Published:** 2012-11-03

**Authors:** Mukut Gohain, Nagarajan Loganathan, Barend C. B. Bezuidenhoudt, Andreas Roodt

**Affiliations:** a Department of Chemistry, University of the Free State, PO Box 339, Bloemfontein 9300, South Africa

## Abstract

In the title compound, C_24_H_15_BrO_3_, the pyran­ochromenone ring is essentially planar, while the 2-bromo­phenyl group is almost perpendicular to it [85.58 (6)°]. In the crystal, inversion dimers linked by pairs of weak C—H⋯π bonds occur; there is also a short inter­atomic contact found between the Br and carbonyl O atoms [3.016 (1) Å].

## Related literature

For coumarin chemistry and applications, see: Hinman *et al.* (1956 ▶); Soine (1964 ▶); Murray *et al.* (1982 ▶); Patil *et al.* (1993 ▶); Verotta *et al.* (2004 ▶); Heide (2009 ▶); Magolan *et al.* (2012 ▶). For related structures, see: Shi *et al.* (2004 ▶, 2005 ▶); Lakshmi *et al.* (2006 ▶). For related synthesis and structures, see: Naveen *et al.* (2007 ▶); Shaabani *et al.* (2008 ▶); Sarma *et al.* (2010 ▶); He *et al.* (2010 ▶).
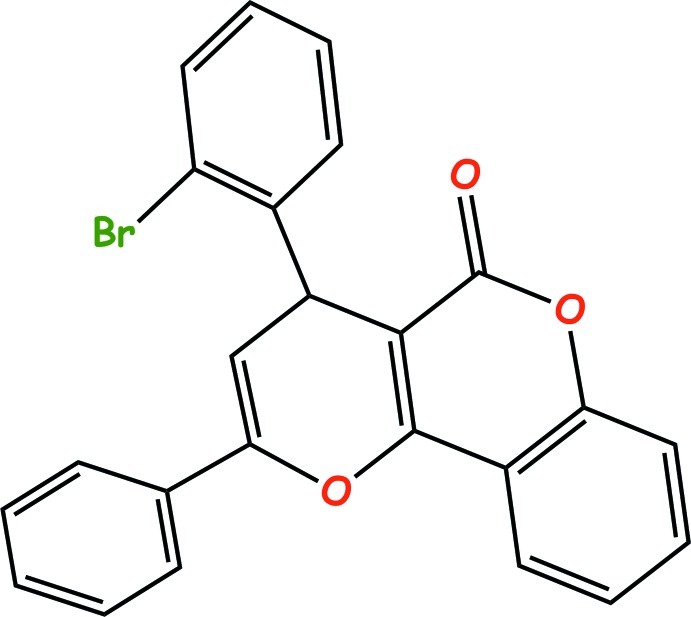


## Experimental

### 

#### Crystal data

C_24_H_15_BrO_3_
*M* *_r_* = 431.27
Monoclinic, 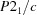

*a* = 11.5959 (2) Å
*b* = 17.7890 (4) Å
*c* = 8.7610 (2) Å
β = 97.060 (1)°
*V* = 1793.53 (7) Å^3^
*Z* = 4
Mo *K*α radiation
μ = 2.32 mm^−1^
*T* = 100 K
0.52 × 0.40 × 0.23 mm

#### Data collection

Bruker X8 APEXII KappaCCD diffractometer
Absorption correction: multi-scan (*SADABS*; Bruker, 2008 ▶) *T*_min_ = 0.380, *T*_max_ = 0.618
41256 measured reflections
4464 independent reflections
4019 reflections with *I* > 2σ(*I*)
*R*_int_ = 0.045

#### Refinement

*R*[*F* ^2^ > 2σ(*F* ^2^)] = 0.027
*wR*(*F* ^2^) = 0.068
*S* = 1.05
4464 reflections
253 parameters
H-atom parameters constrained
Δρ_max_ = 0.51 e Å^−3^
Δρ_min_ = −0.34 e Å^−3^

### 

Data collection: *APEX2* (Bruker, 2008 ▶); cell refinement: *SAINT-Plus* (Bruker, 2008 ▶); data reduction: *SAINT-Plus*; program(s) used to solve structure: *SHELXTL* (Sheldrick, 2008 ▶) and *WinGX* (Farrugia,1999 ▶); program(s) used to refine structure: *SHELXTL*; molecular graphics: *DIAMOND* (Brandenburg & Putz, 2005 ▶); software used to prepare material for publication: *SHELXTL*.

## Supplementary Material

Crystal structure: contains datablock(s) I, global. DOI: 10.1107/S1600536812044510/bv2211sup1.cif

Structure factors: contains datablock(s) I. DOI: 10.1107/S1600536812044510/bv2211Isup2.hkl

Supplementary material file. DOI: 10.1107/S1600536812044510/bv2211Isup3.cml

Additional supplementary materials:  crystallographic information; 3D view; checkCIF report

## Figures and Tables

**Table 1:** Hydrogen-bond geometry (Å, °) *Cg*4 is the centroid of the C12–C17 ring.

*D*—H⋯*A*	*D*—H	H⋯*A*	*D*⋯*A*	*D*—H⋯*A*
C3—H3⋯*Cg*4^i^	1.00	2.80	3.4956 (18)	127

Symmetry code: (i) 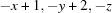.

**Fig. 1.**
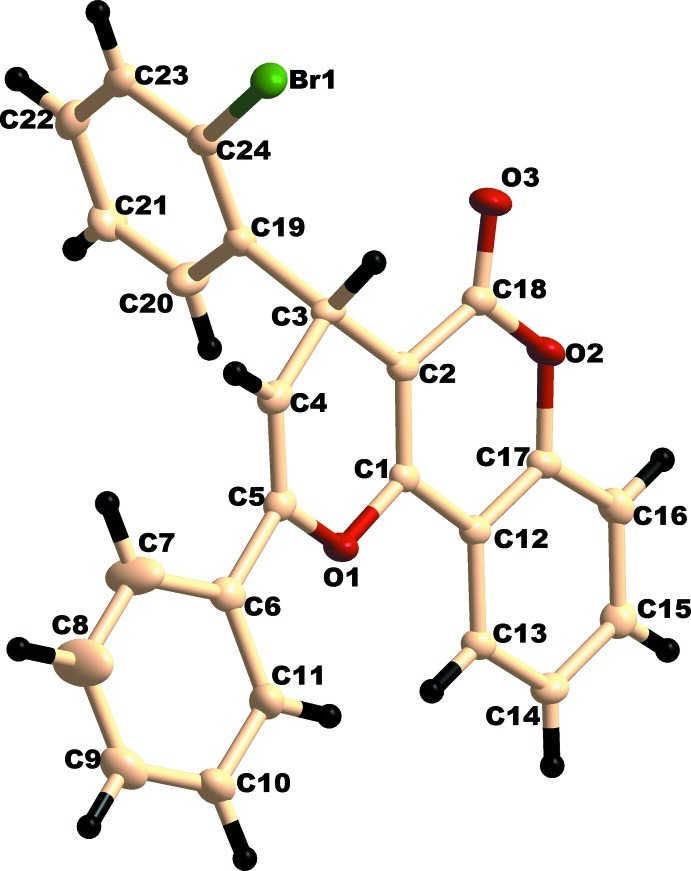
DIAMOND 3.2i picture of the title compound 1 (ellipsoid model with 50% probability).

**Fig. 2.**
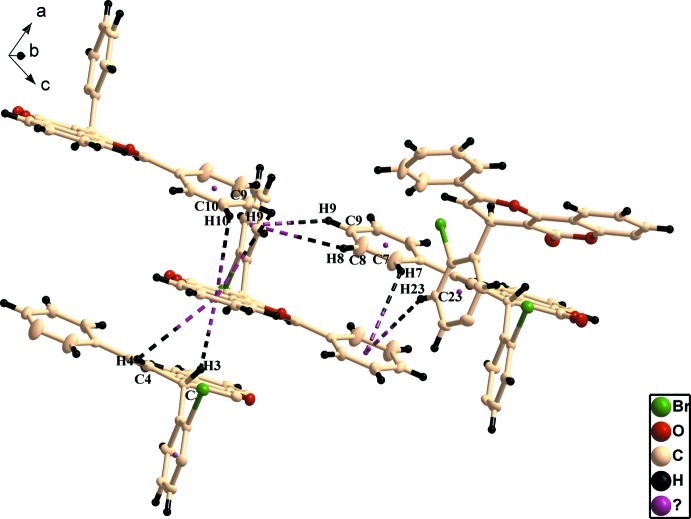
DIAMOND 3.2i picture of 1, showing the intermolecular C—H &bull;&bull;&bull; π interactions. symmetry codes are 2 - x, 0.5 + y, 0.5 - z; 2 - x, -y, 1 - z; x, y, z; 1 - x, -y, -z.

**Fig. 3.**
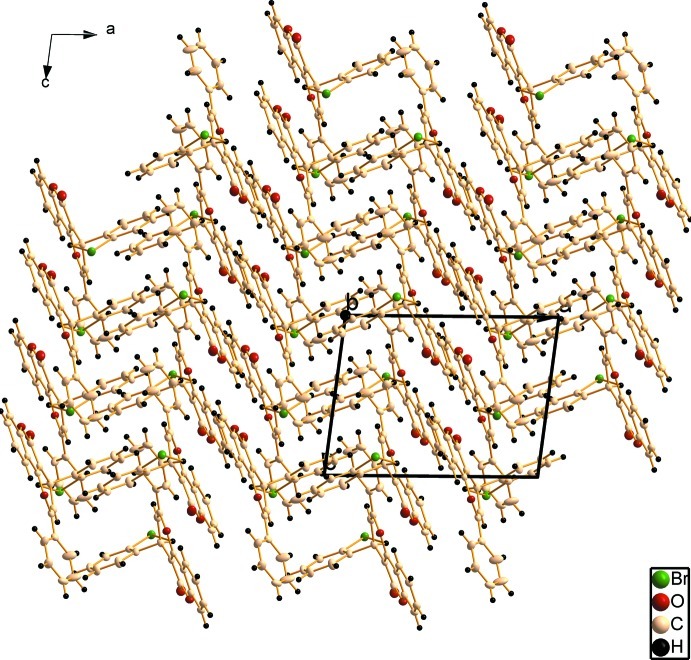
Unit cell packing of the title compound 1 along a-c place (DIAMOND 3.2i representation).

## supplementary crystallographic information

### Comment

Compound 1 is a derivative of a well known phytochemical called coumarin. The derivatives of coumarins are valued for their interesting medicinal applications (Murray *et al.*, 1982; Heide *et al.*, 2009 & Soine *et al.*, 1964). For example, some of them are inhibitors of HIV-1 reverse transcriptase (Patil *et al.*, 1993), while others are being used as anti-bacterial (Verotta *et al.*, 2004), anti-biotic (Hinman *et al.*, 1956) and anti-neoplastic (Magolan *et al.*, 2012) agents. Many authors have synthesized coumarin derivatives using their own approaches (Naveen *et al.*, 2007; Shaabani *et al.*, 2008 & Sarma *et al.*, 2010) and we adapted the synthetic procedure by He *et al.*, 2010. There are also quite a few structures published that are related to compound 1 (Shi *et al.*, 2004; Shi *et al.*, 2005; He *et al.*, 2010 & Lakshmi *et al.*,2006).

Recently He *et al.*, 2010, synthesized various functionalized pyranochromenones and reported the crystal structure of the 2,4-diphenyl pyrano(3,2 - c)chromen-5(4*H*)-one (2). Structurally, both compounds 1 and 2 are quite similar. We adapted their synthetic method for the preparation of 1. Our close examination of the crystal structure 1 (Fig. 1) reveals that the bonds C2—C3 (1.508 (2) Å and C3—C4 (1.506 (2) Å are essentially single bonds. The mean plane analysis of 1 shows the pyranochromen-5(4*H*)-one ring is almost planar. The deviation observed is maximum for the C4 [(0.1166 (17) Å] and C5 [(0.1024 (14) Å] atoms located next to O1 and primary C3 of the pyran ring respectively. The dihedral angle between the chromene (atoms C12 to C17, O2, C18, C1 and C2) and pyrane (C1 to C5 and O1) of pyranochromen-5(4*H*)-one ring is 5.39 (5) °. The phenyl group (atoms C6 to C11) attached to C5 is slightly tilted from the parent plane (dihedral angle is 17.50 (8) ° and the torsion angle of O1—C5—C6—C7 is 157.68 (1) °. The 2-bromophenyl group (C19 to C24 and Br1) connected to C3 is almost perpendicular to the pyranochromen-5(4*H*)-one ring [dihedral angle is 85.58 (6)° and torsion angles of C4—C3—C19—C20 is 81.41 (8) and C4—C3—C19—C24 is 95.90 (1)°].

Several edge-to-face intermolecular C—H ··· π interactions are observed in compound 1 (Fig. 2) and the atomic parameters [distance and angles] are as follows.

a) phenyl ring of the chromenone with C3—H3 [2.804 (1) Å; 146.75 (4)°] and C4—H4 [3.812 (4) Å; 92.71 (4)°] of pyran ring.

b) phenyl ring of the chromenone with C9—H9 [3.798 (4) Å; 84.12 (2)°] and C10—H10 [3.238 (2) Å; 95.65 (3)°] of 2-phenyl group.

c) 2-bromophenyl ring with C9—H9 [3.076 (2) Å; 141.50 (3)°] and C8—H8 [3.652 (5) Å; (115.99 (2)°] of 2-phenyl group.

d) 2-phenyl ring with C23—H23 [3.301 (8) Å; (146.75 (8)°] of 2-bromophenyl group and [C7—H73.639 (4) Å; (128.73 (3)°] of 2-phenyl group.

Strong C—H ··· Br intramolecular interaction is also observed [C3—H3 ··· Br1 2.668 (7) Å; 113.66 (9)°]. The unit cell packing diagram shows a zigzag arrangement of atoms running along the *b* axis (Fig. 3). There is also a short interatomic contact found between Br1 and O3 (3.016 (1)Å).

### Experimental

The synthesis is adapted from the procedure previously published (He *et al.* 2010). A mixture of 4-hydroxycoumarin (0.3 mmol) and 3-(2-bromophenyl)-1-phenylprop-2-en-1-one (0.25 mmol) and 4 Å molecular sieves (0.25 g) were taken in 5 ml of dichloromethane solvent. 2, 3-Dichloro-5,6-dicyano-1,4-benzoquinone (DDQ) (0.5 mmol) was added in portions during 15 min and the reaction mixture were allowed to stir for the 20–30 min. It was then filtered through a Celite plug and purified by column chromatography on silica gel with petroleum ether and ethyl acetate (10:1) as the eluent. The solution was evaporated under vacuo and the pale yellow solid obtained was dissolved in hot acetonitrile. Upon slow evaporation, colourless crystals suitable for X-ray diffraction were obtained. m.p. 238–239°C; Yield. 80%. 1H NMR (600 MHz, CDCl3) δ 8.05 (d, J = 10.3 Hz, 1H), 7.72 (d, J = 7.3 Hz, 2H), 7.68 – 7.53 (m, 2H), 7.49 – 7.39 (m, 5H), 7.32 – 7.07 (m, 2H), 5.91 (d, J = 4.1 Hz, 1H), 5.25 (d, J = 4.0 Hz, 1H). 13 C NMR (150 MHz, CDCl3) δ 161.2(C), 157.3(C), 153.1(C), 146.9(C), 142.5(C), 133.3(CH), 132.6(C), 132.4(CH), 129.7(CH), 129.4(CH), 128.7(2xCH), 128.6(CH), 128.2(CH), 124.8(2xCH), 124.4(CH), 123.4(C), 122.9(CH), 117.1(CH), 114.4(C), 102.4(CH), 102.3(C), 36.5(CH).

### Refinement

The aromatic H atoms were positioned geometrically and allowed to ride on their parent atoms, with *U*_iso_(H) =1.2Ueq(parent) of the parent atom with a C—H distance of 0.93. The methyl H atoms were placed in geometrically idealized positions and constrained to ride on its parent atoms with *U*_iso_(H) = 1.5Ueq(C) and at a distance of 0.96 Å; their torsion angles were optimized from electron density

### Crystal data

**Table:**

C_24_H_15_BrO_3_	*F*(000) = 872
*M**_r_* = 431.27	*D*_x_ = 1.597 Mg m^−^^3^
Monoclinic, *P*2_1_/*c*	Mo *K*α radiation, λ = 0.71073 Å
Hall symbol: -P 2ybc	Cell parameters from 9421 reflections
*a* = 11.5959 (2) Å	θ = 2.6–28.3°
*b* = 17.7890 (4) Å	µ = 2.32 mm^−^^1^
*c* = 8.7610 (2) Å	*T* = 100 K
β = 97.060 (1)°	Cuboidal, colourless
*V* = 1793.53 (7) Å^3^	0.52 × 0.40 × 0.23 mm
*Z* = 4	

### Data collection

**Table:**

Bruker X8 APEXII KappaCCD diffractometer	4464 independent reflections
Radiation source: sealed tube	4019 reflections with *I* > 2σ(*I*)
Graphite monochromator	*R*_int_ = 0.045
φ and ω scans	θ_max_ = 28.3°, θ_min_ = 1.8°
Absorption correction: multi-scan (*SADABS*; Bruker, 2008)	*h* = −15→15
*T*_min_ = 0.380, *T*_max_ = 0.618	*k* = −20→23
41256 measured reflections	*l* = −10→11

### Refinement

**Table:**

Refinement on *F*^2^	Primary atom site location: structure-invariant direct methods
Least-squares matrix: full	Secondary atom site location: difference Fourier map
*R*[*F*^2^ > 2σ(*F*^2^)] = 0.027	Hydrogen site location: inferred from neighbouring sites
*wR*(*F*^2^) = 0.068	H-atom parameters constrained
*S* = 1.05	*w* = 1/[σ^2^(*F*_o_^2^) + (0.0271*P*)^2^ + 1.4892*P*] where *P* = (*F*_o_^2^ + 2*F*_c_^2^)/3
4464 reflections	(Δ/σ)_max_ = 0.003
253 parameters	Δρ_max_ = 0.51 e Å^−^^3^
0 restraints	Δρ_min_ = −0.34 e Å^−^^3^

### Special details

**Table:**

Experimental. Data collected on a Bruker X8 ApexII Kappa CCD diffractometer with 10 s/frame exposure time(total of 2250, width 0.5°)covering up to θ = 28.29° with 99.9% completeness accomplished.
Geometry. All e.s.d.'s (except the e.s.d. in the dihedral angle between two l.s. planes) are estimated using the full covariance matrix. The cell e.s.d.'s are taken into account individually in the estimation of e.s.d.'s in distances, angles and torsion angles; correlations between e.s.d.'s in cell parameters are only used when they are defined by crystal symmetry. An approximate (isotropic) treatment of cell e.s.d.'s is used for estimating e.s.d.'s involving l.s. planes.
Refinement. Refinement of *F*^2^ against ALL reflections. The weighted *R*-factor *wR* and goodness of fit *S* are based on *F*^2^, conventional *R*-factors *R* are based on *F*, with *F* set to zero for negative *F*^2^. The threshold expression of *F*^2^ > σ(*F*^2^) is used only for calculating *R*-factors(gt) *etc*. and is not relevant to the choice of reflections for refinement. *R*-factors based on *F*^2^ are statistically about twice as large as those based on *F*, and *R*- factors based on ALL data will be even larger.

### Fractional atomic coordinates and isotropic or equivalent isotropic displacement parameters (Å^2^)

**Table:**

	*x*	*y*	*z*	*U*_iso_*/*U*_eq_	
Br1	0.771558 (15)	0.721619 (9)	0.11776 (2)	0.02123 (6)	
O1	0.70255 (10)	1.05778 (6)	0.14190 (13)	0.0176 (2)	
O2	0.55197 (11)	0.98739 (7)	−0.28387 (14)	0.0206 (2)	
O3	0.59774 (12)	0.86907 (7)	−0.23016 (16)	0.0266 (3)	
C1	0.65713 (13)	1.03040 (9)	0.00302 (18)	0.0152 (3)	
C2	0.66299 (14)	0.95736 (9)	−0.03718 (19)	0.0170 (3)	
C3	0.72297 (14)	0.89890 (9)	0.0691 (2)	0.0178 (3)	
H3	0.6676	0.8565	0.0783	0.021*	
C4	0.75403 (14)	0.93335 (10)	0.2258 (2)	0.0194 (3)	
H4	0.7808	0.9008	0.3087	0.023*	
C5	0.74635 (14)	1.00623 (9)	0.25547 (19)	0.0175 (3)	
C6	0.77853 (15)	1.04556 (10)	0.40272 (19)	0.0192 (3)	
C7	0.8584 (2)	1.01472 (12)	0.5172 (2)	0.0392 (5)	
H7	0.8900	0.9663	0.5029	0.047*	
C8	0.8922 (2)	1.05422 (13)	0.6525 (2)	0.0445 (6)	
H8	0.9487	1.0332	0.7282	0.053*	
C9	0.84469 (18)	1.12329 (12)	0.6777 (2)	0.0285 (4)	
H9	0.8670	1.1497	0.7709	0.034*	
C10	0.76475 (17)	1.15344 (12)	0.5665 (2)	0.0291 (4)	
H10	0.7309	1.2009	0.5836	0.035*	
C11	0.73243 (16)	1.11577 (11)	0.4291 (2)	0.0263 (4)	
H11	0.6783	1.1382	0.3523	0.032*	
C12	0.60043 (13)	1.08645 (9)	−0.09917 (18)	0.0155 (3)	
C13	0.59292 (14)	1.16273 (9)	−0.06145 (19)	0.0173 (3)	
H13	0.6272	1.1803	0.0361	0.021*	
C14	0.53576 (15)	1.21245 (9)	−0.1660 (2)	0.0191 (3)	
H14	0.5306	1.2641	−0.1404	0.023*	
C15	0.48548 (15)	1.18643 (10)	−0.30964 (19)	0.0200 (3)	
H15	0.4460	1.2208	−0.3809	0.024*	
C16	0.49225 (15)	1.11142 (10)	−0.34978 (19)	0.0200 (3)	
H16	0.4584	1.0940	−0.4477	0.024*	
C17	0.54995 (14)	1.06213 (9)	−0.24295 (19)	0.0171 (3)	
C18	0.60497 (14)	0.93349 (10)	−0.1856 (2)	0.0192 (3)	
C19	0.83245 (14)	0.86731 (9)	0.0112 (2)	0.0189 (3)	
C20	0.90660 (16)	0.91560 (10)	−0.0560 (2)	0.0266 (4)	
H20	0.8870	0.9674	−0.0664	0.032*	
C21	1.00623 (16)	0.89077 (11)	−0.1072 (2)	0.0260 (4)	
H21	1.0541	0.9250	−0.1539	0.031*	
C22	1.03834 (15)	0.81506 (11)	−0.0915 (2)	0.0251 (4)	
H22	1.1076	0.7977	−0.1276	0.030*	
C23	0.96779 (15)	0.76574 (10)	−0.0225 (2)	0.0217 (3)	
H23	0.9890	0.7143	−0.0094	0.026*	
C24	0.86588 (14)	0.79205 (9)	0.02731 (19)	0.0179 (3)	

### Atomic displacement parameters (Å^2^)

**Table:**

	*U*^11^	*U*^22^	*U*^33^	*U*^12^	*U*^13^	*U*^23^
Br1	0.02278 (9)	0.01590 (9)	0.02471 (10)	0.00087 (6)	0.00178 (6)	0.00514 (6)
O1	0.0210 (6)	0.0138 (5)	0.0177 (5)	−0.0002 (4)	0.0007 (4)	−0.0012 (4)
O2	0.0245 (6)	0.0165 (6)	0.0206 (6)	−0.0026 (5)	0.0020 (5)	−0.0052 (5)
O3	0.0293 (7)	0.0169 (6)	0.0336 (7)	−0.0020 (5)	0.0035 (5)	−0.0096 (5)
C1	0.0134 (7)	0.0151 (7)	0.0177 (7)	−0.0021 (6)	0.0045 (6)	−0.0012 (6)
C2	0.0149 (7)	0.0154 (8)	0.0213 (8)	−0.0017 (6)	0.0055 (6)	−0.0016 (6)
C3	0.0175 (7)	0.0113 (7)	0.0257 (8)	−0.0009 (6)	0.0068 (6)	−0.0018 (6)
C4	0.0184 (8)	0.0173 (8)	0.0234 (8)	0.0008 (6)	0.0063 (6)	0.0022 (6)
C5	0.0159 (7)	0.0173 (8)	0.0200 (8)	0.0000 (6)	0.0052 (6)	0.0022 (6)
C6	0.0205 (8)	0.0192 (8)	0.0188 (8)	−0.0035 (6)	0.0062 (6)	0.0009 (6)
C7	0.0645 (15)	0.0242 (10)	0.0264 (10)	0.0119 (10)	−0.0043 (10)	0.0010 (8)
C8	0.0708 (17)	0.0355 (12)	0.0229 (10)	0.0075 (11)	−0.0110 (10)	0.0045 (9)
C9	0.0365 (10)	0.0325 (10)	0.0171 (8)	−0.0095 (8)	0.0061 (7)	−0.0026 (7)
C10	0.0247 (9)	0.0293 (10)	0.0332 (10)	−0.0007 (8)	0.0025 (7)	−0.0124 (8)
C11	0.0218 (9)	0.0263 (9)	0.0292 (9)	0.0029 (7)	−0.0027 (7)	−0.0078 (8)
C12	0.0148 (7)	0.0150 (7)	0.0175 (7)	−0.0016 (6)	0.0053 (6)	−0.0008 (6)
C13	0.0190 (7)	0.0155 (8)	0.0178 (7)	−0.0014 (6)	0.0045 (6)	−0.0009 (6)
C14	0.0219 (8)	0.0143 (8)	0.0219 (8)	−0.0007 (6)	0.0065 (6)	0.0017 (6)
C15	0.0209 (8)	0.0204 (8)	0.0192 (8)	−0.0008 (6)	0.0048 (6)	0.0052 (6)
C16	0.0212 (8)	0.0229 (9)	0.0161 (8)	−0.0041 (7)	0.0039 (6)	−0.0001 (6)
C17	0.0174 (7)	0.0157 (8)	0.0192 (8)	−0.0026 (6)	0.0062 (6)	−0.0026 (6)
C18	0.0176 (8)	0.0169 (8)	0.0240 (8)	−0.0010 (6)	0.0061 (6)	−0.0036 (6)
C19	0.0170 (8)	0.0175 (8)	0.0223 (8)	−0.0001 (6)	0.0030 (6)	−0.0033 (6)
C20	0.0249 (9)	0.0159 (8)	0.0405 (11)	0.0000 (7)	0.0105 (8)	0.0007 (7)
C21	0.0220 (9)	0.0230 (9)	0.0342 (10)	−0.0060 (7)	0.0083 (7)	0.0035 (7)
C22	0.0178 (8)	0.0292 (10)	0.0289 (9)	0.0042 (7)	0.0056 (7)	−0.0035 (7)
C23	0.0211 (8)	0.0210 (9)	0.0226 (8)	0.0046 (7)	0.0004 (7)	−0.0024 (6)
C24	0.0191 (8)	0.0170 (8)	0.0171 (8)	−0.0004 (6)	0.0006 (6)	−0.0015 (6)

### Geometric parameters (Å, º)

**Table:**

Br1—C24	1.8992 (17)	C10—C11	1.388 (3)
O1—C1	1.3555 (19)	C10—H10	0.9500
O1—C5	1.4017 (19)	C11—H11	0.9500
O2—C17	1.378 (2)	C12—C17	1.391 (2)
O2—C18	1.382 (2)	C12—C13	1.402 (2)
O3—C18	1.210 (2)	C13—C14	1.382 (2)
C1—C2	1.350 (2)	C13—H13	0.9500
C1—C12	1.443 (2)	C14—C15	1.399 (2)
C2—C18	1.452 (2)	C14—H14	0.9500
C2—C3	1.508 (2)	C15—C16	1.385 (2)
C3—C4	1.506 (2)	C15—H15	0.9500
C3—C19	1.531 (2)	C16—C17	1.392 (2)
C3—H3	1.0000	C16—H16	0.9500
C4—C5	1.327 (2)	C19—C20	1.396 (2)
C4—H4	0.9500	C19—C24	1.396 (2)
C5—C6	1.475 (2)	C20—C21	1.363 (3)
C6—C11	1.389 (3)	C20—H20	0.9500
C6—C7	1.391 (3)	C21—C22	1.400 (3)
C7—C8	1.392 (3)	C21—H21	0.9500
C7—H7	0.9500	C22—C23	1.388 (3)
C8—C9	1.375 (3)	C22—H22	0.9500
C8—H8	0.9500	C23—C24	1.390 (2)
C9—C10	1.369 (3)	C23—H23	0.9500
C9—H9	0.9500		
			
C1—O1—C5	117.97 (13)	C17—C12—C1	117.11 (14)
C17—O2—C18	121.86 (13)	C13—C12—C1	124.00 (15)
C2—C1—O1	123.61 (15)	C14—C13—C12	120.12 (15)
C2—C1—C12	122.46 (15)	C14—C13—H13	119.9
O1—C1—C12	113.94 (13)	C12—C13—H13	119.9
C1—C2—C18	118.84 (15)	C13—C14—C15	119.80 (15)
C1—C2—C3	122.47 (15)	C13—C14—H14	120.1
C18—C2—C3	118.63 (14)	C15—C14—H14	120.1
C4—C3—C2	108.83 (13)	C16—C15—C14	121.13 (16)
C4—C3—C19	109.64 (14)	C16—C15—H15	119.4
C2—C3—C19	112.78 (14)	C14—C15—H15	119.4
C4—C3—H3	108.5	C15—C16—C17	118.28 (16)
C2—C3—H3	108.5	C15—C16—H16	120.9
C19—C3—H3	108.5	C17—C16—H16	120.9
C5—C4—C3	124.17 (16)	O2—C17—C12	121.13 (15)
C5—C4—H4	117.9	O2—C17—C16	117.06 (15)
C3—C4—H4	117.9	C12—C17—C16	121.79 (15)
C4—C5—O1	121.81 (15)	O3—C18—O2	116.61 (15)
C4—C5—C6	128.24 (16)	O3—C18—C2	124.89 (17)
O1—C5—C6	109.95 (14)	O2—C18—C2	118.49 (14)
C11—C6—C7	118.06 (17)	C20—C19—C24	117.06 (16)
C11—C6—C5	120.72 (16)	C20—C19—C3	119.52 (15)
C7—C6—C5	121.19 (16)	C24—C19—C3	123.37 (15)
C6—C7—C8	120.51 (19)	C21—C20—C19	122.03 (17)
C6—C7—H7	119.7	C21—C20—H20	119.0
C8—C7—H7	119.7	C19—C20—H20	119.0
C9—C8—C7	120.7 (2)	C20—C21—C22	120.34 (17)
C9—C8—H8	119.6	C20—C21—H21	119.8
C7—C8—H8	119.6	C22—C21—H21	119.8
C10—C9—C8	119.04 (18)	C23—C22—C21	119.17 (17)
C10—C9—H9	120.5	C23—C22—H22	120.4
C8—C9—H9	120.5	C21—C22—H22	120.4
C9—C10—C11	120.99 (19)	C22—C23—C24	119.55 (17)
C9—C10—H10	119.5	C22—C23—H23	120.2
C11—C10—H10	119.5	C24—C23—H23	120.2
C10—C11—C6	120.65 (18)	C23—C24—C19	121.83 (16)
C10—C11—H11	119.7	C23—C24—Br1	117.66 (13)
C6—C11—H11	119.7	C19—C24—Br1	120.51 (13)
C17—C12—C13	118.89 (15)		

### Hydrogen-bond geometry (Å, º)

Cg4 is the centroid of the C12–C17 ring.

**Table:**

*D*—H···*A*	*D*—H	H···*A*	*D*···*A*	*D*—H···*A*
C3—H3···*Cg*4^i^	1.00	2.80	3.4956 (18)	127

Symmetry code: (i) −*x*+1, −*y*+2, −*z*.
